# Intravenous versus oral iron supplementation for correction of post-transplant anaemia in renal transplant patients

**DOI:** 10.1186/1471-2369-10-14

**Published:** 2009-06-06

**Authors:** David W Mudge, Ken-Soon Tan, Rhianna Miles, David W Johnson, Scott B Campbell, Carmel M Hawley, Nicole M Isbel, Carolyn L Van Eps, David L Nicol

**Affiliations:** 1 Department of Nephrology, University of Queensland at Princess Alexandra Hospital, Brisbane, Australia; 2 Renal Dialysis Unit, Logan Hospital, Logan, Australia; 3 Department of Renal Medicine, Greenslopes Private Hospital, Brisbane, Australia; 4 Department of Nephrology, University of Queensland at Princess Alexandra Hospital, Brisbane, Australia; 5 Department of Urology, University of Queensland at Princess Alexandra Hospital, Brisbane, Australia

## Abstract

**Background:**

Post-transplant anaemia remains a common problem after kidney transplantation, with an incidence ranging from nearly 80% at day 0 to about 25% at 1 year. It has been associated with poor graft outcome, and recently has also been shown to be associated with increased mortality.

Our transplant unit routinely administers oral iron supplements to renal transplant recipients but this is frequently accompanied by side effects, mainly gastrointestinal intolerance. Intravenous iron is frequently administered to dialysis patients and we sought to investigate this mode of administration in transplant recipients after noticing less anaemia in several patients who had received intravenous iron just prior to being called in for transplantation.

**Methods:**

This study is a single-centre, prospective, open-label, randomised, controlled trial of oral versus intravenous iron supplements in renal transplant recipients and aims to recruit approximately 100 patients over a 12-month period. Patients will be randomised to receive a single dose of 500 mg iron polymaltose (intravenous iron group) or 2 ferrous sulphate slow-release tablets daily (oral iron group). The primary outcome is time to normalisation of haemoglobin post-transplant. Prospective power calculations have indicated that a minimum of 48 patients in each group would have to be followed up for 3 months in order to have a 90% probability of detecting a halving of the time to correction of haemoglobin levels to ≥110 g/l in iron-treated patients, assuming an α of 0.05. All eligible adult patients undergoing renal transplantation at the Princess Alexandra Hospital will be offered participation in the trial. Exclusion criteria will include iron overload (transferrin saturation >50% or ferritin >800 μg/l), or previous intolerance of either oral or intravenous iron supplements.

**Discussion:**

If the trial shows a reduction in the time to correction of anaemia with intravenous iron or less side effects than oral iron, then intravenous iron may become the standard of treatment in this patient group.

## Background

Post-transplant anaemia (PTA) remains a common problem after kidney transplantation, with an incidence ranging from nearly 80% at day 0 to about 25% at 1 year [1-4]. It has been associated with poor graft outcome, and recently has also been shown to be associated with increased mortality [5]

PTA may occur at any time after renal transplantation. Early PTA (post surgery until 3 months) is most likely to be related to pre-transplant anaemia, the surgery itself, iron deficiency, immunosuppression and infection [6,7]. PTA is more likely to be related to consequences of long-term immunosuppression or a failing graft. All the large scale studies to date have focused mainly on late PTA [4,6,7], and there is a lack of studies looking at the management of early PTA, in particular, the optimal management of iron deficiency.

Our unit routinely administers oral ferrous sulphate to all post transplant patients, however this is not without problems including gastrointestinal intolerance, and interference with the absorption of immunosuppressant medications by leading to significant constipation.

Recently, we have observed that several dialysis patients serendipitously given intravenous iron for treatment of iron deficiency anaemia immediately prior to transplantation, have had better Hb levels in the post-transplant period, without needing oral iron supplementation. In a retrospective case series, Gillespie and Symonds used intravenous ferrous gluconate to treat 15 paediatric and young adult renal transplant recipients and found that doses of up to 250 mg induced a statistically significant increase in mean haemoglobin levels. There were only 4 adverse events reported in 3 patients, all self limiting and none life threatening [8]. However, there are no trials to date comparing the efficacy of intravenous versus oral iron replacement.

We surmise that giving a single dose of intravenous iron (as ferrous gluconate – Ferrosig^®^) may be superior to a protracted course of oral iron.

The aim of the study therefore, is to compare the time to normalisation of Hb post-transplant in patients given a single dose of IV iron as compared with oral iron.

## Methods

### Patients

All adult patients receiving a renal transplant at the Princess Alexandra Hospital will be invited to participate in the study. Informed consent will be obtained from all participants. The study protocol has been approved by the Princess Alexandra Hospital Human Research Ethics Committee (HREC), approval number 2007/142.

Inclusion criteria will include new living-donor or deceased-donor renal transplant recipients aged 18 years or over who are able to give written informed consent. Exclusion criteria will include iron overload (transferrin saturation >50% or ferritin >800 μg/l), women lactating, pregnant or of child-bearing potential not using a reliable contraceptive method, patients with a history of psychological illness or condition which interferes with their ability to understand or comply with the requirements of the study, patients who have received a new investigational drug within the last 4 weeks, and intolerance of intravenous or oral iron supplements.

### Design

This is an open-label, randomised, controlled clinical trial in which the primary outcome measure will be the mean time to resolution of PTA (defined as Hb ≥110 g/l). Patients meeting the inclusion criteria and consenting to participate in the study will be randomly allocated in a 1:1 ratio to either (a) oral iron (ferrous sulphate slow-release 2 tablets mane – the most common current practice in the transplant unit) or (b) intravenous iron polymaltose as a 500 mg single dose given within the first 5 days after transplantation. Randomisation will occur by the use of sequentially numbered, sealed, opaque envelopes with stratification for calcineurin inhibitor type (cyclosporin or tacrolimus).

Immunosuppressive therapy will be managed according to the unit's standard protocols. Patients will have blood tests checked as per the unit's follow up protocol for recent transplant recipients. We will collect full blood count, renal function, and calcineurin inhibitor trough levels on a weekly basis, as well as iron studies, B12, and folate at one monthly intervals.

### Power and statistical analysis

Prospective power calculations have indicated that a minimum of 48 patients in each group would have to be followed up for 3 months in order to have a 90% probability of detecting a halving of the time to correction of haemoglobin levels to ≥110 g/l in iron-treated patients, assuming an α of 0.05.

### Adverse events during the investigation

Each patient will be observed closely during the period of the study looking for adverse events and if any occur they will be documented on a Case Report Form. Any significant adverse events will also be notified to the P.A. Hospital Research Ethics Committee.

Gastrointestinal adverse effects will be defined as the onset of nausea, vomiting, abdominal cramping or diarrhoea. Infusion related reactions will be described as self-limiting flushing sweating, chills, myalgias, arthralgias, bronchospasm and chest pain occurring at the time of the infusion. All infectious episodes and the results of subsequent microbiological investigation will be recorded during the study period.

## Discussion

As far as the authors are aware, this will be the first randomised trial of intravenous versus oral iron supplementation for PTA in kidney transplantation. If the trial shows a reduction in the time to correction of anaemia with intravenous iron or alternatively if there is no difference but fewer side effects than oral iron, then intravenous iron may become the preferred treatment in this patient group.

## Competing interests

The authors declare that they have no competing interests.

## Authors' contributions

DM conceived the study, edited the ethics submission, secured the funding and oversaw the running of the trial. KST wrote the ethics submission and supervised recruitment of patients. RM recruited patients. CH performed the statistical power calculations. DJ, SC, NI, CVE and DN all assisted in the trial design and protocol. All authors read and approved the final manuscript.

## Pre-publication history

The pre-publication history for this paper can be accessed here:

http://www.biomedcentral.com/1471-2369/10/14/prepub
